# Intracellular Ca^2+^ is important for flagellin-triggered defense in Arabidopsis and involves inositol polyphosphate signaling

**DOI:** 10.1093/jxb/erx176

**Published:** 2017-06-08

**Authors:** Yi Ma, Yichen Zhao, Gerald A Berkowitz

**Affiliations:** Department of Plant Science and Landscape Architecture, Agricultural Biotechnology Laboratory, University of Connecticut, Storrs, CT, USA

**Keywords:** Ca^2+^ signaling, G-proteins, immune responses, inositol polyphosphate, intracellular Ca^2+^ store, PAMP

## Abstract

Cytosolic Ca^2+^ increase is a crucial and early step of plant immunity evoked by pathogen-associated molecular patterns (PAMPs) such as flagellin (flg). Components responsible for this increase are still not uncovered, although current models of plant immune signaling portray extracellular Ca^2+^ influx as paramount to flg activation of defense pathways. Work presented here provides new insights into cytosolic Ca^2+^ increase associated with flg-induced defense responses. We show that extracellular Ca^2+^ contributes more to immune responses evoked by plant elicitor peptide (Pep3) than that evoked by flg, indicating an intracellular Ca^2+^ source responsible for immune responses evoked by flg. Genetic impairment of the inositol polyphosphate (InsP) and G-protein signal associated with flg perception reduced flg-dependent immune responses. Previous work indicates that prior exposure of Arabidopsis plants to flg leads to an immune response reflected by less vigorous growth of a pathogenic microbe. We found that this immune response to flg was compromised in mutants lacking the ability to generate an InsP or G-protein signal. We conclude that the recruitment of intracellular Ca^2+^ stores by flg may involve InsP and G-protein signaling. We also found a notable difference in contribution of intracellular stores of Ca^2+^ to the immune signaling evoked by another PAMP, elf18 peptide, which had a very different response profile to impairment of InsP signaling. Although Ca^2+^ signaling is at the core of the innate immune as well as hypersensitive response to plant pathogens, it appears that the molecular mechanisms generating the Ca^2+^ signal in response to different PAMPs are different.

## Teaser

The perception of a pathogen leads to the recruitment of intracellular Ca^2+^ pools associated with inositol polyphosphate, which contributes to a signal transduction cascade leading to immune responses.

## Introduction

Detection of invading pathogens by plant cells is typically initiated upon binding of evolutionarily conserved essential components of the microbes, namely pathogen associated molecular patterns (PAMPs), to pattern recognition receptor (PRR) proteins localized on the plasma membrane. Pathogen perception (upon PAMP–PRR interaction) initiates a signal transduction cascade in the cytosol of plant cells that leads to basal immune responses, known as PAMP-triggered immunity (PTI). An early and paramount step of this immune signaling cascade is a transient elevation of cytosolic Ca^2+^. This Ca^2+^ elevation is critical to downstream signaling and initiation of defense responses that limit spread of the pathogen. One of the most well-characterized PAMP–PRR signaling pathways is the interaction of the bacterial motility organ protein flagellin (flg) with the flg receptor FLS2 (Boller and Felix, 2009; Monaghan and Zipfel, 2012). Recent studies (Aslam *et al.*, 2008; Segonzac and Zipfel, 2011; Kwaaitaal *et al.*, 2011) have led to the inference that the cytosolic Ca^2+^ elevation and resulting Ca^2+^-dependent immune signaling that occurs downstream from the epitope-containing peptide flg22 interaction with the FLS2 receptor (flg22:FLS2 from hereon) is entirely or primarily dependent on influx of extracellular Ca^2+^ into the cytosol through plasma membrane-localized Ca^2+^-conducting ion channels. This conclusion is primarily based on experiments with Arabidopsis leaves or seedlings exposed to EGTA, which is known to chelate extracellular Ca^2+^. These studies also used Ca^2+^ channel blockers and other inhibitors.

Our recent studies employed several alternative experimental approaches to evaluate the dependence of flg22:FLS2-initiated immune responses on movement of extracellular Ca^2+^ into the cytosol. Working with purified Arabidopsis leaf mesophyll protoplasts, we observed a maximal degree of Ca^2+^-dependent immune signaling initiated by exposure of protoplasts to flg22 in the presence or absence of Ca^2+^ in the external solution (Ma *et al.*, 2012). This result suggested that flg22:FLS2-initiated Ca^2+^-dependent immune signaling could be occurring through a signal transduction system that mobilizes intracellular Ca^2+^ pools for export to the cytosol. The vacuole is a substantial (estimated at 1–10 mM, similar to the cell wall; Mariani *et al.*, 2003) internal reservoir of Ca^2+^ available for export across the tonoplast. We speculated that one component of flg22:FLS2-dependent immune signaling involves the generation of a cytosolic secondary messenger molecule that activates Ca^2+^ efflux across the tonoplast to the cytosol.

Further support for this possibility was obtained using Arabidopsis plants expressing mammalian type I inositol polyphosphate 5-phosphatase (IP5-ptase); this enzyme specifically catabolizes inositol 1,4,5-trisphosphate (IP3) in animals (Laxminarayan *et al.*, 1993; Majerus *et al.*, 1999). The IP5-ptase Arabidopsis plants display impaired *myo*-inositol polyphosphate (InsP) generation and cytosolic Ca^2+^ elevations in response to external signals (Perera *et al.*, 2008). InsPs are cytosolic secondary messengers that mobilize vacuolar pools of Ca^2+^ during some signaling cascades (Munnik and Nielsen, 2011). We found that disruption of InsP generation (in IP5-ptase plants) had a greater effect on flg22-dependent cytosolic Ca^2+^ elevation than on cytosolic Ca^2+^ elevation activated by a different plasma membrane PRR known to act on plasma membrane-localized Ca^2+^ channels (Qi *et al.*, 2010; Ma *et al.*, 2012). More recent work provides additional support for the involvement of InsP generation in PTI and defense cascades activated by at least some PAMPs such as flg22. Murphy *et al.* (2008) showed that impaired hexakisphosphate (IP6) biosynthesis (IP3 is a precursor) in Arabidopsis *ipk1* (InsP5 2-kinase, catalysing the final step of IP6 biosynthesis) and *ips* (catalysing the initial step in IP6 biosynthesis) null mutants led to increased susceptibility to different types of pathogens. Hung *et al.* (2014) found that exposure to the virulent pathogen *Pseudomonas syringae* pv tomato (Pst) DC 3000 led to an elevation in IP3 in Arabidopsis seedlings.

These results, with protoplasts exposed to Ca^2+^-free solutions in one case and using IP5-ptase-expressing plants in a second experimental approach suggested that mobilization of intracellular Ca^2+^ pools may be a significant component of immune cascades responding to at least some PAMPs. Not much is known about the molecular components of the signaling pathway linking flg22 binding to the FLS2 receptor, and the resulting downstream generation of the Ca^2+^ signal ‘vital’ (see Ranf *et al.*, 2011) to initiation of immune responses. Genetic approaches have documented that flg22-dependent cytosolic Ca^2+^ elevation does not involve the plasma membrane-localized (Ali *et al.*, 2007) Ca^2+^ channel CNGC2 (Jeworutzki *et al.*, 2010; Ma *et al.*, 2012), while inhibitor studies suggest that a Ca^2+^-conducting glutamate receptor may be involved in generating the Ca^2+^ signal evoked by this PAMP (Kwaaitaal *et al.*, 2011). In the work reported here, we extend our prior studies to further characterize some of the molecular steps of PAMP-induced immune defense responses in Arabidopsis plants. The focus of the work was on the role of InsP in Ca^2+^-mediated responses to the bacterial PAMP flg, and the pathogen *P. syringae.*

## Materials and methods

### Plant material

All Arabidopsis lines used in the reported work are in the Columbia (Col) background. The *ipk1* T-DNA null insertional mutant (corresponding to locus At5g42810) was first identified by Stevenson-Paulik *et al.* (2005) and phenotypically characterized as having low levels of IP6 and increased sensitivity to pathogens by Murphy *et al.* (2008). Seeds of the *ipk1* (SALK_065337C) mutant were obtained from the Arabidopsis Biological Resource Center and G-protein mutant seeds *gpa1-4*, *agb1-2*, *xlg2-1*, and *xlg3-1* (xlg, extra large G-protein; allele numbers are omitted in later description) were obtained from the Sarah Assmann lab at Pennsylvania State University. *ips1* and *ips2* mutant seeds were obtained from the Alex M. Murphy lab at the University of Cambridge (Murphy *et al.*, 2008). Provenance and our use of Col-aeq and IP5-ptase-aeq genotypes have been reported in our prior publications (Ma *et al.*, 2012). The aequorin-expressing line ‘Col-aeq’ was used as a wild type (WT) control for comparisons of cytosolic Ca^2+^ changes, NO generation, gene expression and mitogen-activated protein kinase (MPK) phosphorylation with IP5-ptase-aeq plants (Perera *et al.*, 2008)—shortened as ‘IP5-ptase’ throughout this paper except where clarification of the presence of the aequorin gene was necessary. Seeds were surface sterilized and spread on Petri dishes containing half-strength Murashige and Skoog (MS) salts (Caisson Labs, Logan, UT, USA), MES (adjusted to pH 5.7 with Tris), 1% sucrose, and 1% agar, and grown in a growth chamber with a day (80–100 μmol m^−2^ s^−1^ illumination)/night cycle of 12/12 h at 25 °C. For the experiments involving measurement of cytosolic Ca^2+^, seeds were grown on square plates containing ½ MS medium for 10 d and the seedlings were used for measurement. For the NO experiment, seeds were transplanted to soil about 10 d after germination. Seedlings were transplanted into pots containing LP5 soil-less mix containing starter fertilizer (Sun Gro) at 12 h light (100 μmol m^−2^ s^−1^ illumination)/12 h dark (72% relative humidity) and 22 °C. Seeds were stratified at 4 °C in the dark for 3 d before use. During growth, plants were irrigated with Jack’s Professional Peat-Lite 20:10:20 (NP_2_O_5_-K_2_O) solution (at 2 g l^−1^) one to two times to provide supplementary fertilizer.

### Ligand and reagent concentrations

The preponderance of prior studies examining flg22 signaling in Arabidopsis have used the PAMP at or near 1 µM to activate defense responses (e.g. Aslam *et al.*, 2008; Schulze *et al.*, 2010; Ranf *et al.*, 2011; Kwaaitaal *et al.*, 2011; Frei dit Frey *et al.*, 2012; Cao *et al.*, 2013) even though this bacterial peptide evokes basal defense responses in Arabidopsis at much lower concentrations (e.g. Jeworutzki *et al.*, 2010). When flg22 was used as the sole activating ligand in our experiments, the peptide was used at 1 µM final concentration (as noted in figure legends) so that our results can be compared with the bulk of the work that has examined the flg22-dependent immune response. Alternatively, in a few of our experiments, the effects of flg22 on immune signaling was compared with those evoked by Pep3; our prior work characterizing Pep signal transduction used the ligand at a much lower level (20 nM) (Qi *et al.*, 2010; Ma *et al.*, 2012, 2013). Thus, for the work reported here, we used flg22 at 1 µM when it was used alone in experiments. For experiments that compared effects of Pep and flg22, both activating peptide ligands were used at 20 nM; these concentrations are noted in appropriate figure legends. The bacterial peptide PAMP elf18 was used for some experiments. This peptide was used at 20 nM for measurement of cytosolic Ca^2+^ elevation, and for monitoring gene expression. Effects of elf18 on ROS production used ~2-week-old seedlings and followed the luminol luminescence methods of Ranf *et al.* (2011), where 1 µM elf18 was used.

The aminopolycarboxylic acids BAPTA and EGTA are exclusively extracellular Ca^2+^ chelating agents (Polisensky and Braam, 1996). When used at a high enough concentration, they restrict Ca^2+^ entry into the cytosol by reducing the pool of free apoplastic Ca^2+^ in equilibrium with the approximately millimolar level of the cation associated with the negative changes of the cell wall. EGTA and BAPTA were used in our studies at 10 mM. Use of the Ca^2+^ chelating agents at this concentration range has been shown in numerous prior studies to impair plant cell functions dependent on influx of extracellular Ca^2+^ (e.g. Jeter *et al.*, 2004; Aslam, *et al.*, 2008; Segonzac and Zipfel, 2011).

### Cytosolic Ca^2+^ measurements

The method of Qi *et al.* (2010) was used with slight modification for cytosolic Ca^2+^ measurements using aeq-expressing plants. Individual (10-day-old) seedlings were placed in a capless 2 ml centrifuge tube containing 300 μl distilled water. For each tube, 0.6 μl coelenterazine-cp (CTZ-cp, AAT Bioquest Inc.) was added (l0 μM final concentration in 0.2% (v/v) ethanol). Seedlings were incubated at room temperature in the dark overnight to allow CTZ incorporation into plants. As the CTZ-cp is a light-sensitive reagent, all preparatory steps after adding the CTZ-cp were carried out in dark; tubes were covered with foil paper.

A single-tube luminometer (TD-20/20, Promega) was used for measurement of the luminescence level. The centrifuge tubes were placed in the luminometer, and left for 2–3 min to allow seedlings to recover from touch-induced Ca^2+^ spikes. The luminescence level was measured every second and ligand (flg22) was added only after background luminescence of the leaves was stable; 300 μL flg22 (2 µM, dissolved in distilled water at 2× concentration) was added to the tubes containing seedling by gently pipetting the solution against the interior wall of the centrifuge tube. For BAPTA and EGTA pre-treatment, 250 μl 40 mM BAPTA or EGTA, with a final concentration of 10 mM, was added to the solution and incubate for 30 min prior to adding flg22. After the incubation, 250 μl 80 nM flg22 or Pep3 was added to a final concentration of 20 nM. Results shown in the figures are mean values calculated from a minimum of at least three biological replicates. After recording luminescence from a treatment replicate, the remaining aeq (i.e. not bound to Ca^2+^) in an assay tube was discharged by adding 800 μl Ca^2+^ release buffer (2 M CaCl_2_.6H_2_O in 30% ethanol) with continued recording for ~10 min or until the instantaneous luminescence level dropped below 2000. Values obtained from aeq discharge were used to convert luminescence readings to cytosolic Ca^2+^ concentration for each treatment replicate using an algorithm as described by Qi *et al.* (2010).

### NO production in guard cells

The method for NO detection in guard cells using the NO-specific fluorescent dye diaminofluorescein-2 diacetate (DAF-2DA; Invitrogen) was adapted from Ali *et al.* (2007). Rosette leaves of 4- to 6-week-old WT, *ipk1* and IP5-ptase transgenic plants were detached from plants and used to make epidermal peels. Epidermal peels were incubated in buffer A (10 mM KCl, 25 mM MES-KOH, and 0.1 mM CaCl_2_.6H_2_O, pH 6.15) for 1–2 h. Peels were then placed in 3 ml buffer B (NO synthase reaction kit buffer; Sigma-Aldrich) which contained 50 µM DAF-2DA to load cells with NO-specific dye. After 30 min incubation to load the NO-specific dye in the tissue, peels were washed three times in buffer A and then incubated in 3 ml buffer B containing ligand flg22 (1 µM) for 5 min. The epidermal peels were placed underneath a coverslip on a microscope slide with several drops of buffer B. NO-dependent fluorescence was monitored over time. Mean maximal fluorescence of guard cells (occurring ~5–10 min after ligand addition) in a peel was ascertained exactly as described previously (Ma *et al.*, 2009) and averaged to generate one biological replicate. After washing in buffer A and transfer to buffer B, when no ligand was present in buffer B leaf tissue remained dark and showed no fluorescence. Maximal fluorescence measurements of approximately three guard cells in an epidermal peel were averaged for one replicate of a ligand treatment. For each treatment, captured images at the maximum fluorescence intensity for treatment replicates were used to calculate data represented as means. Quantitative analyses of the NO-dependent fluorescence in guard cell pairs were undertaken using ImageJ as described in Ali *et al.* (2007). The digitized image showing maximum fluorescence for guard cell pairs from an epidermal peel represented a genotype replicate; a minimum of three epidermal peels were analysed for each genotype.

### qPCR analysis

Growth of Arabidopsis seedlings in half-strength MS liquid medium was performed according to Ma *et al.* (2012). The seedlings were grown in separate tubes containing 3 ml liquid medium for 14 d on a shaker (180 rpm) with 24 h illumination (~90 mol m^–2^ s^–1^) at 22 °C. flg22 (1 µM) was added directly to the growth medium and the seedlings were incubated for 30 min. Water was given to the seedlings for the control treatment. For BAPTA and EGTA pre-treatment, WT seedlings were fully submerged and incubated in 10 mM BAPTA or EGTA for 30 min, then flg22 or Pep3 was added to a final concentration of 20 nM, and the seedlings were treated for a further 30 min for gene induction. After incubation, the seedlings were collected and frozen immediately with liquid N_2_, and stored at −80 °C for future use. Total RNA was extracted using the Plant RNA Extraction Kit (Macherey-Nagel, 740955).

After extraction, 500 ng to 2 μg of total RNA was used for reverse transcription using the High Capacity cDNA Reverse Transcription Kit (Applied Biosystems). The synthesized cDNA was diluted 1:10 (v/v) in water, and 1 µl of the diluted cDNA was used for each qPCR reaction. qPCR assays were performed using the Applied Biosystems 7900 HT real-time PCR system. Treatment effects on the expression level of *WRKY29* (AT4G23550) and *WRKY33* (AT2G38470) were examined. *UBQ10* (AT4G05320) was used as an endogenous control. The primers used for these analyses were as follows: *WRKY29* (forward) 5′-ATCCAACGGATCAAGAGCTG-3′, *WKRY29* (reverse) 5′- GCGTCCGACAACAGATTCTC-3′; *WRKY33* (forward) 5′-CCCGTTGGCTTCATACCG T-3′, *WRKY33* (reverse) 5′-TTACAGCGAGTGTCAATTTGGC-3′; and *UBQ10* (forward) 5′-CGGCTACCACATCCAAGGAA-3′, *UBQ10* (reverse) 5′-GCTGGAATTACCGCGGCT-3′. We used the qPCR assay for the FRK1 gene (Bio-Rad).

For each analysis, three technical replicates were tested on one plate, and the mean value of each treatment was generated from analysis of at least three biological replicates from RNA isolated from different individual seedling. ANOVA of corresponding threshold cycle (*C*_T_) values was used to generate standard errors of the means for control treatments (Schmittgen and Livak, 2008). In many cases, qPCR analysis of gene expression involved the evaluation of flg22 effects on different genotypes. In these cases, gene expression in the water control was normalized to 1 for each genotype in a given experiment.

### MPK phosphorylation

The MPK phosphorylation assay was adapted from the method described by Keinath *et al.* (2010). Seedling growth is the same as that for qPCR. flg22 at 1 µM and the control solvent (water) were added directly into each well. The seedlings were kept in the medium with the treatment for 0, 5, 15, and 30 min. Seedlings were then blotted dry and frozen in liquid nitrogen. For time point ‘0 min’, seedlings were immediately moved out of the assay well, dried and frozen in liquid nitrogen after the ligand was added into the medium. For protein extraction of the seedlings, seedlings were ground with 1 ml of extraction buffer (10 mM HEPES, pH 7.5, 100 mM NaCl, 1 mM EDTA, 10% glycerol, 0.5% Triton X-100 and protease inhibitor cocktail (Roche) (Lu *et al.*, 2010).

Extracted protein was added to 6× sodium dodecyl sulfate (SDS) dye, and denatured by incubation at 95 °C for 5 min. The proteins were loaded onto a 12% SDS-PAGE gel. After electrophoresis, proteins were transferred from the gel to a nitrocellulose membrane. After being blocked with 5% bovine serum albumin (BSA; Research Products International), the membrane was incubated with the primary antibody anti-phospho-p44/42 MAPK (Erk1/2) (Thr202/Tyr204) (Cell Signaling Technology, used at 1:1000 dilution in 5% (w/v) BSA, 1× Tris-buffered saline (TBS), 0.1% Tween 20) at 4 °C overnight. After incubation, the membrane was washed with 1× TBS with 0.1% Tween three times. The membrane was then incubated with secondary antibody, anti-rabbit IgG (GE Healthcare Life Sciences, used at 1:5000 dilution in 5% (w/v) BSA, 1× TBS, 0.1 % Tween 20) at room temperature for 1 h.

Chemiluminescent horseradish peroxidase substrates (ECL reagents, Thermo Scientific) were used in the detection of western blots. The chemiluminescent substrate was added to the membrane and incubated for 1 min. The extent of MPK phosphorylation was detected by exposure of photographic X-ray film (Research Products International) to the transfer membranes for 5 min in darkness.

### Growth of virulent *Pseudomonas syringae* in plants

The following method for pre-exposure of plants to flg22 and subsequent inoculation of leaves with virulent pathogen was adapted from Ma *et al* (2013). flg22 (or water as a control) was syringe injected into (attached) rosette leaves from WT, *ipk1* and *gpa1agb1* (in earlier experiments, we used *gpa1agb1* double mutants suggested and provided by Allan Jones at University of North Carolina, Chapel Hills) plants 24 h prior to bacterial inoculation. Pst DC3000 cells were isolated from overnight liquid cultures and resuspended in sterile 10 mM MgCl_2_ to a concentration of 3 × 10^8^ cfu. The resuspended bacteria were applied (in 0.01% (v/v) Silwet L-77 surfactant) by spraying on leaves previously treated with water or flg22 from 4- to 5-week-old plants. Leaf tissue was collected for measurement of bacterial titer 3 d post-inoculation. For each biological replicate, 0.7-cm-diameter leaf discs were cut from leaves of two different plants and ground together in 100 µl of 10 mM MgCl_2_ in a 1.5 ml microcentrifuge tube. The samples were thoroughly vortexed in 900 µl of water and diluted 1:10 serially. Then 10 µl of the samples (and serial dilutions) was spread on plates containing low-salt Luria-Bertani agar medium containing 100 mg l^−1^ rifampicin. Plates were left at 25°C for 3 d; after this incubation period, colonies on plates were counted.

### Use of different plant materials for various immune signaling assays

This report describes results of experiments using a range of plant materials: (i) intact, whole ~2-week-old seedlings, (ii) epidermal peels of mature leaves, and (iii) discs cut from fully expanded, non-senescing leaves. The rationale for the use of these materials is as follows. Most studies of the immune response signal transduction pathway use seedlings (Ranf *et al.*, 2011; Kwaaitaal *et al.*, 2011). Unless there was a specific need to do otherwise, we followed this convention, using seedlings for monitoring effects of PAMP ligands on gene expression, cytosolic Ca^2+^ elevation, and protein phosphorylation. We use the well-studied (Melotto *et al.*, 2006; Ali *et al.*, 2007; Ma *et al.*, 2013) model system of intact guard cells in leaf epidermal peels for NO assays. The mesophyll is destroyed during peel preparation, leaving intact guard cells that can be exposed to solutions containing PAMP ligands. Studies of plant immune responses that affect pathogen growth *in planta* necessarily require monitoring growth of pathogen in intact leaves (Ma *et al.*, 2013); we used this system to monitor immune activation by pre-exposure of leaves to PAMPs prior to inoculation with pathogenic bacteria.

## Results

### Intracellular Ca^2+^ store release contributes to flg22 signaling

**Fig. 1.**
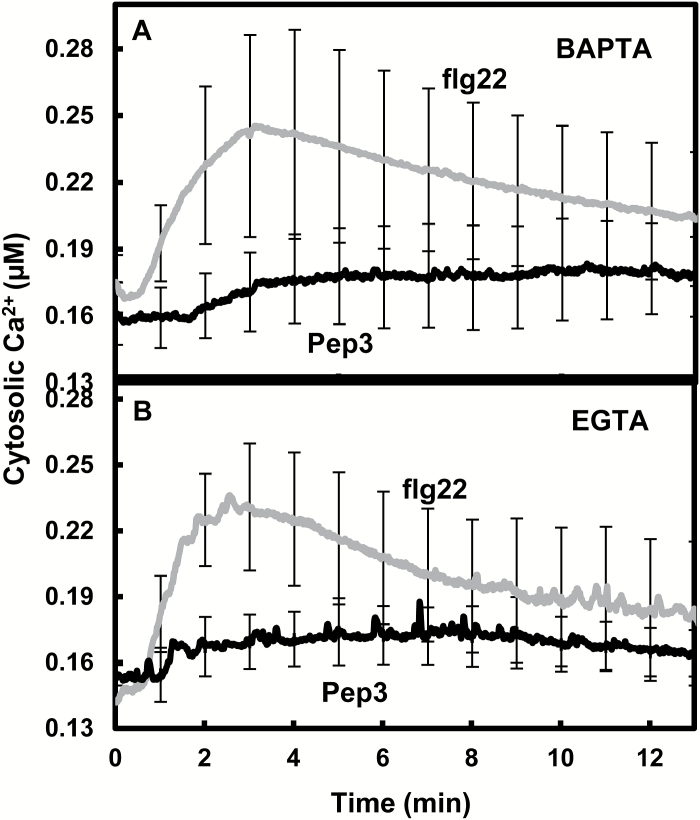
The cytosolic Ca^2+^ elevation in response to flg22 addition is greater than that in response to Pep3 when extracellular Ca^2+^ is limited. Extracellular Ca^2+^ was lowered in these experiments by addition of 10 mM BAPTA (A) or EGTA (B). Pep3 or flg22 ligand (20 nM) was added following 30 min pretreatment of WT (Col-aeq) seedlings with BAPTA or EGTA. Results are presented as mean values of cytosolic Ca^2+^ ±SE (*n*=3).

In a fashion similar to the PAMP flg22, the endogenous plant signaling peptide Pep3 binds to a plasma membrane-localized leucine-rich repeat receptor-like kinase, and initiates a cytosolic Ca^2+^ elevation that leads to immune responses (Qi *et al.*, 2010). However, prior work from this lab (Ma *et al.*, 2012) suggested that the signal transduction pathways downstream from Pep3 and flg22 were different. Pep3-evoked Ca^2+^ signaling was suspected to rely only on extracellular Ca^2+^ in contrast to signaling downstream from flg22 (Ma *et al.*, 2012). We extended this comparison of flg22 and Pep3 signaling in the work shown in Fig. 1A, B. Ca^2+^ signaling evoked by flg22 was compared with that caused by the Pep3 peptide. In this comparison, the two peptides were applied at the same low (20 nM) concentration (see ‘Materials and methods’ for summary of ligand concentrations used in all experiments). Exposure of seedlings to this same level of either immune-activating ligand flg22 or Pep3 in the absence of extracellular chelation agents (EGTA or BAPTA) caused similar levels of cytosolic Ca^2+^ elevation as shown in Supplementary Fig. S1 at *JXB* online; the Ca^2+^ signal generated by 20 nM Pep3 was at least as great as that occurring in response to a similar concentration of flg22. Chelation of extracellular Ca^2+^ with either BAPTA (Fig. 1A) or EGTA (Fig. 1B) was found to have a greater effect on Pep3-dependent Ca^2+^ signal than that evoked by flg22. In the presence of either BAPTA or EGTA, flg22 evoked a much greater Ca^2+^ signal than equivalent levels of Pep3.The results suggest that flg22 signaling includes the activation of intracellular Ca^2+^ stores and in this case, to a greater extent than Pep3.

**Fig. 2.**
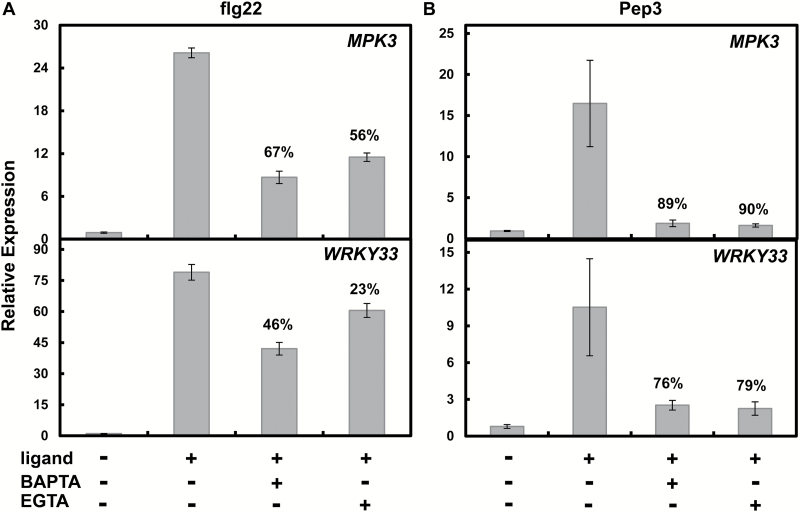
Effects of extracellular Ca^2+^ chelation on (A) flg22- or (B) Pep3-dependent *MPK3* and *WRKY33* expression in WT (Col) seedlings. Peptide ligands (flg22 or Pep3) were used at 20 nM. BAPTA and EGTA were used at a concentration of 10 mM. Three independent experiments were conducted. Results are presented as means±SE. Numbers above some of the bars represent the % inhibition of ligand-dependent gene expression due to addition of the chelating agent. In the absence of added ligand (i.e. without flg22 or Pep3), relative expression of genes was negligible in the absence (first bar of each panel) or presence (not shown) of BAPTA or EGTA. For each of the four panels shown in this figure, significance of means separation between ligand (flg22 or Pep3)-induced gene expression in the absence or presence of chelation (BAPTA or EGTA) was evaluated using ANOVA (Student’s paired *t*-test). In all cases, the expression level in the presence of EGTA or BAPTA was significantly different (*P*<0.05) from the expression level in the absence of a chelating agent. Statistical tests of separation of means that are shown in Figs 3B, 4, 5, 7 and 9 used similar analyses.

Basal immune responses activated by PAMP perception-dependent cytosolic Ca^2+^ elevation during PTI include the increased expression of genes encoding pathogen defense-related transcription factors such as *WRKY33* and *WRKY29*, mitogen-activated protein kinases (MPKs) such as *MPK3*, and receptor-like kinases (RLKs) such as *FRK1* (Asai *et al.*, 2002; Yamaguchi *et al.*, 2010; Ma *et al.*, 2012). Monitoring expression of these genes is a particularly convenient ‘read-out’ assay of immune signal transduction because their expression level is affected relatively quickly (and robustly) by PAMP application, the immune pathway controlling their expression is affected by Ca^2+^ signaling, and these immune defense-associated genes typically respond to a variety of PAMPs (Ranf *et al.*, 2011; Ma *et al.*, 2012, 2013). In the experiments shown in Fig. 2, we compared the effect of chelation of extracellular Ca^2+^ (by application of EGTA or BAPTA to the incubation medium) on defense gene expression induced by flg22 and Pep3. In a fashion similar to the effects of extracellular Ca^2+^ on the generation of the Ca^2+^ signal, the signal transduction cascade downstream from flg22 and Pep3 leading to activation of defense gene expression was also affected differently. The Pep3-dependent expression of *MPK3* and *WRKY33* was inhibited to a much greater extent by application of EGTA or BAPTA (Fig. 2B) than the effects of these extracellular chelating reagents on flg22-induced defense gene expression (Fig. 2A). Along with the results shown in Fig. 1, the experiments shown in Fig. 2 suggest that, unlike Pep3 signaling, flg22 immune signaling involves activation of intracellular Ca^2+^ pools; the signal transduction pathway is not as dependent as Pep3 signaling on extracellular Ca^2+^.

### InsP signals are involved in flg22-triggered innate immune responses

**Fig. 3.**
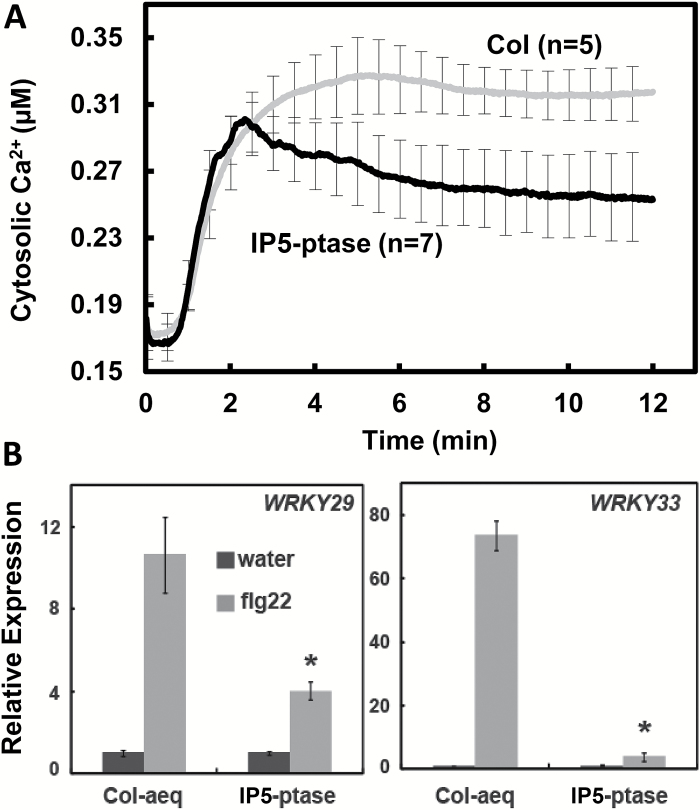
IP3 signaling is involved in flg22-induced Ca^2+^ rise and defense gene upregulation. (A) Exogenous flg22-dependent elevation of cytosolic Ca^2+^ in WT (Col-aeq) and IP5-ptase-aeq seedlings. Ligand (1 µM flg22) was added at time ‘0’; results are presented as mean values of cytosolic Ca^2+^ concentration (replicate number in parentheses) ±SE shown at 0.5 min intervals. Results shown in this figure are compilations from numerous biological replicates. Recordings from individual seedlings indicate that the Ca^2+^ elevation is transitory (e.g. Supplementary Fig. S2). (B) IP3 is involved in flg22-dependent *WRKY29* and *WRKY33* expression. Seedlings were incubated with ligand flg22 (light bars) or water addition (dark bars) for 30 min prior to RNA extraction. Results shown are means±SE (*n*=4) of transcript levels normalized to the level in WT (Col-aeq) seedlings in the absence of flg22. Asterisks indicate significant difference (*P*<0.05) between WT (Col-aeq) and IP5-ptase here seedlings treated with flg22.

IP3 is known to be involved in intracellular Ca^2+^ signaling in animal cells. However, no IP3 receptor (or other InsP receptors) has been identified in plant cells. In order to study IP3 signaling in plants, we used transgenic plants expressing the animal IP5-ptase; as mentioned above, the IP5-ptase in these plants breaks down IP3 generated during signaling cascades in plant cells. Results shown in Fig. 3A demonstrate that impairment of the ability to generate a signal-induced IP3 elevation in plant cells reduces the magnitude of the Ca^2+^ signal generated by flg22. The results in Fig. 3A are consistent with the possibility that IP3-dependent release of intracellular Ca^2+^ stores may contribute to the early and ‘vital’ Ca^2+^ signal generated upon flg22 perception during immune defense responses.

We used flg22-dependent expression of *WRKY29* and *WRKY33* to interrogate the involvement of InsP signaling with basal immunity signaling pathways. We found that InsP signaling was involved in the up-regulation of these transcription factors in response to flg22 perception. The increased expression of *WRKY29* and *WRKY33* that occurred when flg22 was applied to WT plants was impaired in IP5-ptase plants (Fig. 3B).

**Fig. 4.**
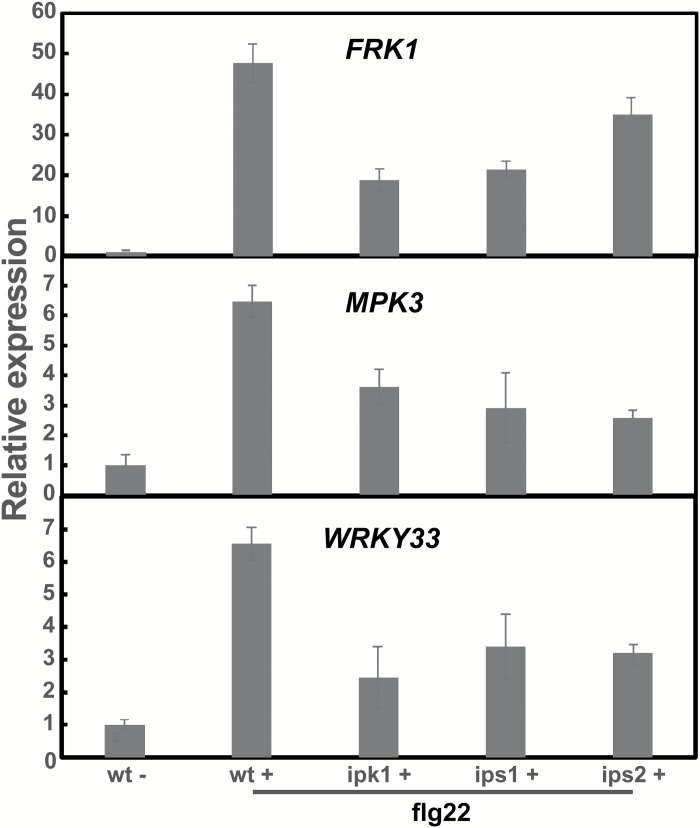
Genetic impairment of InsP generation affects flg22-dependent expression of defense genes. *FRK1*, *MPK3*, and *WRKY33* expression were monitored in Col (WT), *ipk1*, *ips1*, and *ips2* mutant seedlings after addition of flg22. Results shown are means±SE (*n*=3) of transcript levels normalized to the level in WT seedlings treated with water (‘WT–’). Asterisks indicate a significant difference (*P*<0.05) between WT and a mutant genotype in the presence of flg22.

IP3 is a precursor of IP6. The impairment of IP3 levels in IP5-ptase transgenic plants also affects IP6 production (Murphy *et al.*, 2008). In subsequent work, we used additional genetic tools to document the involvement of InsP signaling in flg22-evoked immune responses. T-DNA insertion mutants of *IPK1*, *IPS1*, and *IPS2* were examined. In the same study, IPS mutants *ips1* and *ips2* had lowered IP6 levels from those found in WT seedlings. Our results showed that, as compared with expression in WT seedlings, flg22-dependent expression of *WRKY33*, *MPK3*, and another pathogen-responsive gene, *FRK1*, were reduced around 50% in *ipk1*, *ips1*, and *ips2* null mutants (Fig. 4). The results further support the involvement of InsP signaling in transcriptional modulation associated with flg22-dependent PTI.

Nitric oxide (NO) has been known to be involved in the plant immune response for some time, both as an antimicrobial (Boccara *et al.*, 2005) and in immune signal transduction cascades (Ali *et al.*, 2007; Boller and Felix, 2009). Some work has also shown that NO is generated specifically in response to flg22 perception. Melotto *et al.* (2006) demonstrated NO generation in guard cells responding to flg22 and Zhang *et al.* (2008) later developed a model of (Arabidopsis) guard cell responses to both flg22 and abscisic acid that had NO and cytosolic Ca^2+^ elevation as major nodes in these signaling networks. We recently demonstrated that NO is generated after flg22 elicitation in a FLS2-dependent manner, and used genetic approaches to link NO generation to flg22-evoked, Ca^2+^-dependent immune signaling in Arabidopsis plants.

**Fig. 5.**
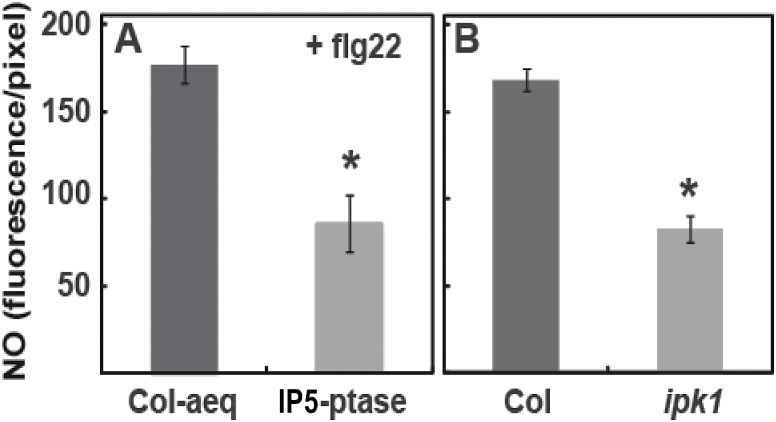
InsP (IP3 and/or IP6) signaling is involved in flg22-induced NO generation. Results are shown for experiments comparing NO generation in (A) Col-aeq and IP5-ptase here plants and (B) Col and *ipk1* null mutant plants. Signals shown (as bars) are means (±SE) of four replicates prepared from leaves of different plants. Fluorescence of approximately three guard cell pairs in an epidermal peel was averaged for one replicate. The maximum fluorescence intensity that could be recorded from the peels was 256 fluorescence units per pixel. Significant differences (at *P*<0.05) between fluorescence recorded from the genotypes used for an experiment are indicated by an asterisk above a bar.

Here, we extend our studies of flg22-dependent NO generation to evaluate the involvement of the secondary messenger molecule(s) InsP in this signaling pathway. As mentioned above, we found that impairment of InsP signaling (in IP5-ptase-expressing plants) resulted in a reduction in flg22-dependent Ca^2+^ elevation (Fig. 3). In the experiment shown in Fig. 5, we found that flg22-dependent generation of NO was similarly affected in IP5-ptase plants (Ma *et al.*, 2013).

InsP signaling is initiated through the generation of IP3 by the activity of the highly regulated enzyme phosphoinositide-specific phospholipase C (PI-PLC). IP5-ptase hydrolyses IP3, and therefore prevents the activation of plant cell processes that respond to this cytosolic signal (Perera *et al.*, 2008). It has been several decades since InsP signaling was shown to play a role in many plant cell responses to environmental and developmental cues. Early studies provided experimental results consistent with the idea that IP3 was the specific polyphosphoinositide that acted in these signaling pathways. More recent work (reviewed in Munnik and Nielsen, 2011) points to IP6, generated downstream from IP3 in the InsP signaling cascade initiated by PI-PLC, as the biologically significant InsP signaling moiety in plants. Generation of IP3 during a signaling cascade could lead to the production of IP6 through the action of IPK1 and 2 (Munnik and Nielsen, 2011). Electrophysiological studies employing isolated plant cell vacuoles have shown that application of either IP3 (Alexandre and Lassalles, 1990; Allen and Sanders, 1995) or IP6 (Lemtiri-Chlieh *et al.*, 2003) to the cytosolic face of the tonoplast results in outward Ca^2+^ currents from the vacuole lumen. Consistent with these patch clamp studies of Ca^2+^ currents across the tonoplast, release of caged IP3 (Lemtiri-Chlieh *et al.*, 2003) or IP6 (Gilroy *et al.*, 1990) in intact protoplasts results in cytoplasmic Ca^2+^ elevation due to release of Ca^2+^ from intracellular stores. Plants lack an InsP receptor akin to the IP3 receptor of animal cells that facilitates the release of internal stores of Ca^2+^ (Boss and Im, 2012). The lack of a *bona fide* receptor leads to obvious questions about the mechanism and molecular identity of plant cell components that link InsP to Ca^2+^ signaling. However, these aforementioned studies do provide strong experimental evidence that InsP elevation does lead to a rise in cytosolic Ca^2+^ in plant cells by activation of an endomembrane channel.

Here, we used plants expressing IP5-ptase as well as the *ipk1* mutant as genetic tools to probe InsP signaling related to PAMP perception. As shown in Fig. 5, flg22-dependent generation of the pathogen-response signaling molecule NO was impaired in *ipk1* null mutants as compared with the response of wild type (WT) plants to flg22. The extent of impairment of NO generation (as compared with corresponding control plants) was about the same (approximately 50% reduction from levels found in WT plants) in the IP5-ptase and *ipk1* genotypes (Fig. 5). Use of these two different genetic tools in the experiments shown in Fig. 5 indicates that InsP signaling is involved in the generation of NO in response to perception of the PAMP flg22.

**Fig. 6.**
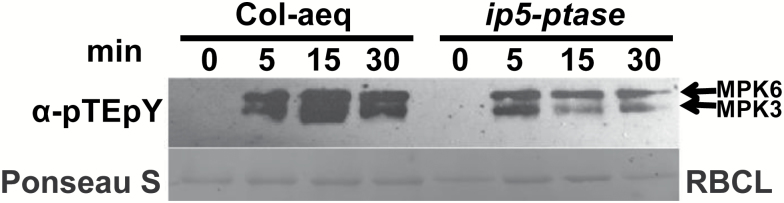
Genetic impairment of InsP signaling affects flg22-induced MPK phosphorylation. Seedlings were treated with 1 μM flg22 at time ‘0’, and were flash frozen in liquid nitrogen 5, 15, and 30 min after treatment. Samples were subjected to immunoblot analysis using antibodies against α-phospho-p44/p42 (α-pTEpY). The Col-aeq WT line was used as an appropriate control for IP5-ptase seedlings, which also express aeq. The lower panel shows RBCL staining as the loading control.

Additional results demonstrating involvement of PI-PLC in the signaling pathway linking PAMP perception with immune responses in plant cells are shown in Fig. 6. MPK signaling cascades (specifically MPK3 and MPK6) are known to be involved in a number of immune responses, including ROS generation and defense gene activation (Zheng *et al.*, 2006; Meng and Zhang, 2013). MPK activation and MPK-dependent steps in immune response signaling cascades are downstream from PAMP-induced cytosolic Ca^2+^ elevation. Impairment of flg22-induced cytosolic Ca^2+^ elevation reduces MPK activation (Ranf *et al.*, 2011; Kwaaitaal *et al.*, 2011). Here, we evaluated the extent of MPK activation state by monitoring MPK phosphorylation as was done in these prior studies. We found that in Arabidopsis genotypes that are impaired in InsP signaling (IP5-ptase-expressing plants), flg22-dependent MPK activation was reduced as compared with that in a corresponding control line (Col-aeq) (Fig. 6). We used *ipk1* mutants, which also have impaired InsP signaling, for a similar experiment as that shown in Fig. 6. In the case of the *ipk1* genotype, we used Col as an appropriate control. Results (not shown) were similar to that shown in Fig. 6; immunoblot analysis indicated a reduction in MPK activation coincident with impairment of InsP signaling (in this case, due to *ipk1* null mutation).

### G-proteins and immune responses

**Fig. 7.**
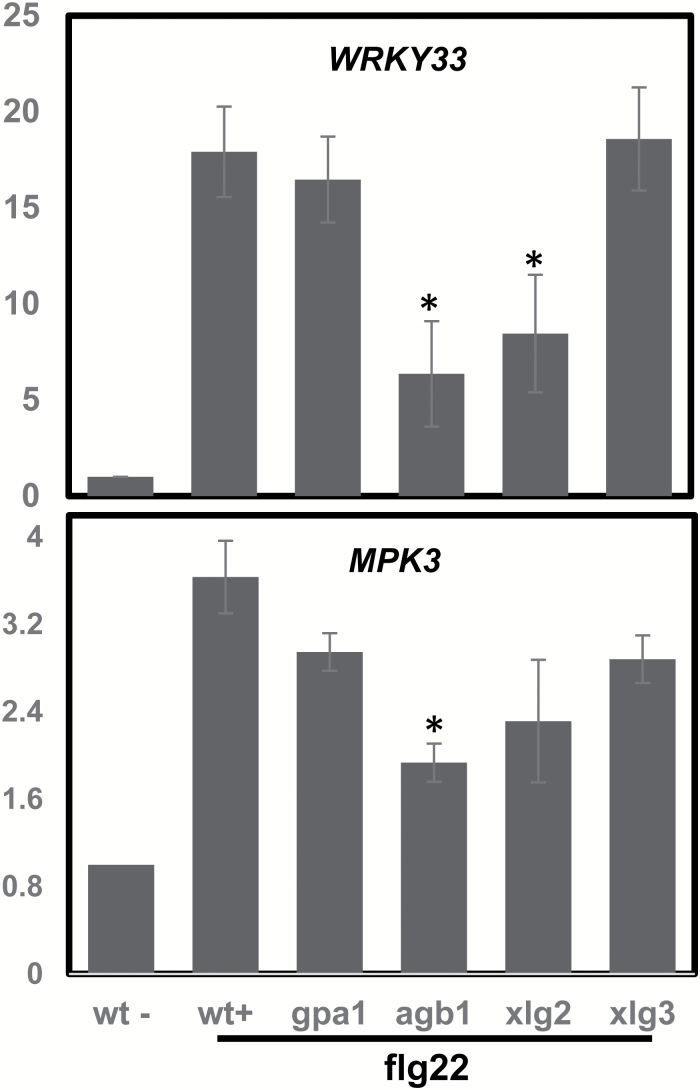
Effects of defective G-protein signaling on flg22-dependent *WRKY33* and *MPK3* expression. Gene expression was monitored in Col (WT), *gpa1*, *agb1*, *xlg2*, and *xlg3* mutant seedlings after addition of flg22. Results shown are means±SE of transcript levels normalized to the level in WT seedlings in the absence of flg22. Asterisks indicate significant difference (*P*<0.05) between WT and a mutant treated with flg22.

Heterotrimeric G-proteins are present in plants and are involved in signaling pathways. In our studies, we associated G-proteins with flg22 signaling. In our evaluation of InsP involvement in basal immune responses, we included the G-protein mutants *gpa1*, *agb1*, *xlg2*, and *xlg3* in some of our experiments. As shown in Fig. 7, the flg22-dependent induction of *WRKY33* was significantly reduced in *agb1* and *xlg2* seedlings compared with WT, while *gpa1* and *xlg3* contained similar amount of *WRKY33* transcripts as WT. Similarly, *MPK3* expression was significantly decreased in *agb1* seedlings compared with WT. The findings indicate that it is not *GPA1* but *AGB1* and *XLG2* that are responsible for G-protein effects on flg22-induced defense gene expression. Recent reports have shown that GPA1 does not play a role in flg22-induced defense signaling (Liu *et al.*, 2013; Maruta *et al.*, 2015; Liang *et al.*, 2016). Instead, AGB1 and XLG2 can form G-protein complexes *in vivo* to contribute to flg22-induced immunity (Maruta *et al.*, 2015; Liang *et al.*, 2016). XLG2 also interacts directly with FLG2 (Liang *et al.*, 2016). Our results are consistent with these previous findings.

### Do different bacterial PAMPs have different signaling pathways?

**Fig. 8.**
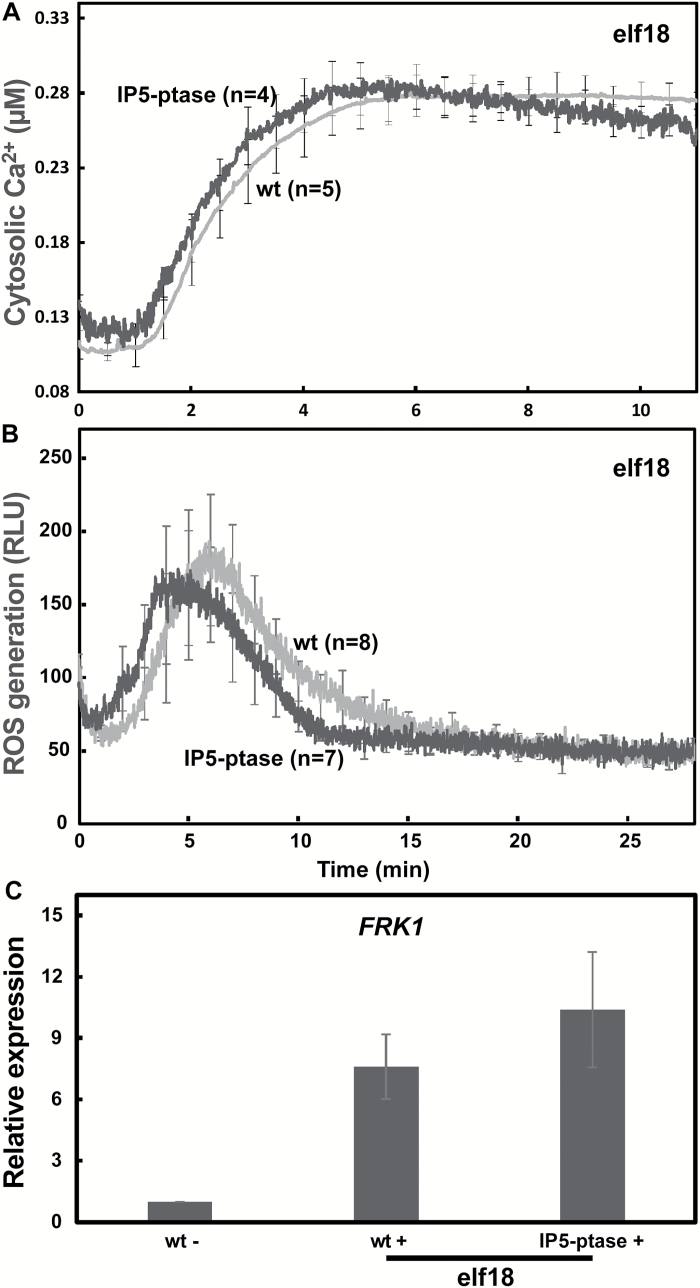
PTI downstream from the PAMP elf18 does not involve InsP signaling. (A) Cytosolic Ca^2+^ elevation was monitored in WT-aeq (*n*=5) and IP5-ptase-aeq seedlings (*n*=4) after elf18 addition at time ‘0’. (B) elf18-triggered ROS production in WT (*n*=8) and IP5-ptase (*n*=7) seedlings (ligand added at time ‘0’). (C) elf18-dependent *FRK1* expression in WT and IP5-ptase seedlings. Results are shown as means±SE (*n*=3) of transcript levels normalized to the level in WT seedlings treated with water.

We found that genetic impairment in the presumed ability of plants to activate the InsP pathway has a significant effect on the immune signal transduction initiated by perception of the bacterial PAMP flg22; Ca^2+^ elevation, defense gene expression, and anti-microbial molecule (nitric oxide) production were all impaired (Figs 1–5). We extended our analysis of InsP signaling involvement in PTI by examining the immune responses activated by another bacterial PAMP, elf18 (which binds to a different receptor from flg22; Boller and Felix, 2009), in WT and IP5-ptase plants (Fig. 8). Some studies have identified specific molecules involved in immune response signaling cascades, including cell membrane coreceptors and cytoplasmic receptor-like kinases both upstream and downstream of Ca^2+^ elevation that are shared by the defense pathways initiated by flg22, elf18 and the DAMP Pep peptides (Roux *et al.*, 2011; Kadota *et al.*, 2014; Ranf *et al.*, 2014; Sreekanta *et al.*, 2015). Most reviews present a model of PTI initiated by elf18 and flg22 as essentially similar (Newman *et al.*, 2013), although some differences in the signal transduction scheme downstream from the two PAMPs have been noted (Shi *et al.*, 2013). Here, we observed markedly different contributions of InsP signaling to the flg22 and the elf18 immune response cascades. In contrast to results with flg22 (Figs 1–4), we found essentially no significant impairment in elf18-dependent Ca^2+^ elevation (Fig. 8A), defense molecule (in the case of elf18, we monitored ROS) generation (Fig. 8B), and defense gene expression (Fig. 8C) in IP5-ptase plants as compared with WT.

### InsP is required for flg22 triggered immunity to virulent pathogens

**Fig. 9.**
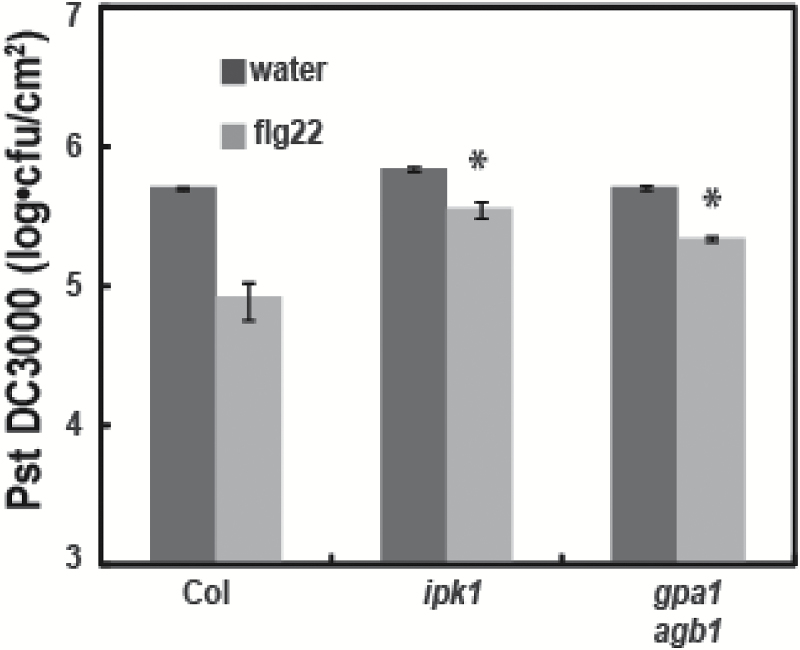
Flg22-induced immunity to a virulent pathogen is compromised in *ipk1* and *gpa1agb1* mutant plants. Proliferation of (virulent) Pst DC3000 was examined on leaves of WT (Col), *ipk1*, and *gpa1agb1* mutant plants pretreated with water (control) or flg22. Plants were pretreated 1 d prior to inoculation with Pst and bacterial growth was evaluated 3 d after inoculation. Results shown (note log scale of ordinate axis) are mean values of Pst recovered from leaves (*n*=4) ±SE. An asterisk above a bar representing bacterial growth in leaves of either *ipk1* or *gpa1agb1* genotypes indicates that for that genotype, bacterial growth in leaves subjected to flg22 pretreatment was significantly different (at *P*<0.05) from the level found in WT leaves subjected to flg22 pretreatment.

The desired biological outcome from immune signaling is an enhancement of host system fitness in the face of a pathogenic challenge. The paramount downstream effect of pathogen defense signaling cascades that are activated by receptor FLS2 perception of the PAMP flg22 is a decrease in pathogen virulence (i.e. a reduction in pathogen proliferation within the plant). Thus, ‘immunization’ of plant leaves by exposure to flg22 could be observed in the experimental results presented in Fig. 9. With WT plants, growth of the (virulent) bacterial pathogen Pst DC3000 (measured 3 d after inoculation) was reduced from (log units) 5.7 to 4.9 when plants were pre-exposed to flg22 24 h prior to inoculation with DC3000 pathogen. Flg22 ‘immunization’ resulted in an 84% reduction in pathogen proliferation. The effectiveness of flg22 to immunize the plant against pathogen growth was compromised when a similar immunization/inoculation protocol was used with either *ipk1* or *gpa1agb1* mutants (Fig. 9). The results of the experiment shown in Fig. 9 support the conclusion that both G-protein signaling and InsP-dependent mobilization of vacuolar Ca^2+^ stores contribute to the immune defense responses activated by flg.

## Discussion

The experimental results in this report provide new information about PAMP-triggered immunity, specifically with regard to the signaling cascade evoked upon perception of the PAMP flg. Chelation of apoplastic Ca^2+^ reduces the Ca^2+^ signal and Ca^2+^-dependent immune signaling occurring in response to Pep3 to a greater extent than effects on flg22 signaling. These results suggest that intracellular Ca^2+^ stores contribute significantly to flg immune signaling. InsP signaling was linked to flg22-dependent generation of a cytosolic Ca^2+^ signal as well as immune defense responses downstream from the Ca^2+^ signal. The cytosolic InsP signaling cascades contributed to flg22-dependent NO generation, MPK activation, and increased expression of pathogen defense genes.

The causal link between the reduction of the flg22-dependent Ca^2+^ signal and an impairment of all of these downstream defense responses is well established in an extensive body of published literature (e.g. Chinchilla *et al.*, 2007; Gust *et al.*, 2007; Aslam *et al.*, 2008; Kwaaitaal *et al.*, 2011; Ma *et al.*, 2013; Ranf *et al.*, 2014). IP6 is downstream from IP3 generation in the InsP signaling cascade. In the case of PTI, impairment of IP6 generation appears to be as effective as impairment of IP3 generation in terms of reducing Ca^2+^-dependent immune responses. Cytosolic IP6 is known from prior work to activate tonoplast-localized Ca^2+^-conducting channels and facilitate vacuolar Ca^2+^ release, leading to elevation of cytosolic Ca^2+^. Our results suggest that impairment in the ability of plants to generate IP3 during signaling cascades affects immune signaling evoked by flg22 through the downstream inhibition of IP6 production. This is apparently not a general phenomenon of PTI. Comparative studies with another bacterial PAMP, the peptide elf18, did not reveal involvement of InsP signaling cascade—a situation more similar to that of Pep peptide signaling.

G-proteins have been associated with both immune responses and InsP signaling in plants (Legendre *et al.*, 1992; Rajasekhar *et al.*, 1999; Zhang *et al.*, 2008; Ishikawa, 2009; Zhu *et al.*, 2009; Jin *et al.*, 2013). Some studies do not support the involvement of G-proteins in specifically the flg22 signaling pathway (Tanaka *et al.*, 2013) while other studies do show impairment of flg22 activation of pathogen defense responses in G-protein subunit null mutants (Ishikawa, 2009; Lorek *et al.*, 2013). Here, we find that impairment of G-protein signaling affects PTI in a fashion similar to what occurs when InsP signaling is inhibited. In both cases (impairment of InsP or G-protein signaling), the pathogen defense signaling cascade that allows for perception of flg leading to an immune response is compromised and pathogen growth in the plant is increased.

**Fig. 10.**
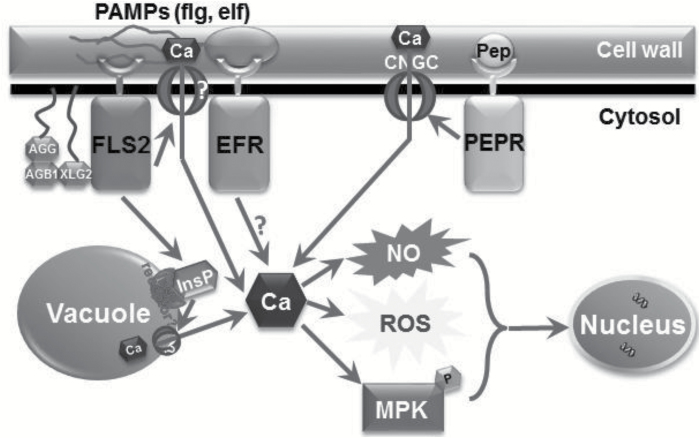
A model depicting the involvement of InsP and G-protein signaling in flg evoked plant innate immunity. PAMPs and Pep bind to plasma membrane localized cognate PRR receptors and triggers cytosolic Ca^2+^ elevation. In the flg:FLS2 signaling pathway, flg-induced full Ca^2+^ elevation requires InsP-dependent Ca^2+^ release from the vacuole. The Ca^2+^ conducting channel on the tonoplast facilitating this conductance is not known. Additionally, previous findings indicate an extracellular Ca^2+^ influx mediated by an unidentified plasma membrane Ca^2+^-conducting channel. G-proteins AGB1 and XLG2 are involved in flg evoked innate immune signaling, in which AGB1 and AGG form the G-protein complex with XLG2, which is also physically associated with FLS2. In contrast, Pep-dependent Ca^2+^ elevation is primarily sourced from apoplastic Ca^2+^ that is mediated through a plasma membrane CNGC channel. Molecular mechanisms responsible for Ca^2+^ elevation induced by another PAMP elf are still unrevealed, but the pathway is different from that induced by either flg or Pep. Although there is specificity for the mechanisms facilitating cytosolic Ca^2+^ elevation, both PAMP and Pep triggered signaling cascades share similar downstream events such as NO and ROS generation, MPK phosphorylation and defense gene activation, eventually leading to basal defense.

G-protein regulation of PI-PLC-dependent InsP generation clearly must involve molecular steps that are different in plants and animals (Urano *et al.*, 2013). However, the work of Jin *et al.* (2013) supports the idea that G-proteins can positively affect plant InsP signaling pathways. This G-protein activation of PI-PLC-dependent InsP (i.e. IP3 and then, downstream, IP6) generation might occur indirectly through some as-yet-unidentified intermediate steps. For example, G-protein subunits have recently been shown to physically and functionally interact with the flg22 receptor FLS2 (Liang *et al.*, 2016). We acknowledge, however, that the work presented in this report does not specifically link G-protein signaling to InsP-induced mobilization of intracellular Ca^2+^ stores during pathogen defense response cascades. A proposed model (Fig. 10) illustrates the involvement of InsP and G-protein in flg-evoked defense signaling pathway. The model also depicts the differences between flg, Pep and elf signaling, which are puzzles that need to be solved in future studies.

IP6 accumulation in food and forage crop plants is known to negatively impact human nutrition as well as environmental quality. Thus, reduction in IP6 levels in crop plants is an important agronomic quality trait that is the target of current bio-engineering efforts. The work in this report reinforces (see discussion in Murphy *et al.*, 2008) that care should be taken to identify specific pools of IP6 that can be manipulated in crop plants without compromising plant immune responses to crop pathogens.

## Supplementary data

Supplementary data are available at *JXB* online.

Fig. S1. flg22- and Pep3-dependent cytosolic Ca^2+^ elevation in WT (Col-aeq) seedlings.

Fig. S2. The flg22-dependent cytosolic Ca^2+^ elevation is transitory.

## Supplementary Material

Supplementary Figures S1-S2
