# Supplementary material for: Intracellular Ca2+ is important for flagellin-triggered defense in Arabidopsis and involves inositol polyphosphate signaling
Source: J Exp Bot. 2017 Jun 8;68(13):3617–28. doi: 10.1093/jxb/erx176 (PMC5853439; doi:10.1093/jxb/erx176)
Supplement: Supplementary Figures S1-S2 [file erx176_suppl_supplementary_figures_s1-s2.pdf]

Fig. S1

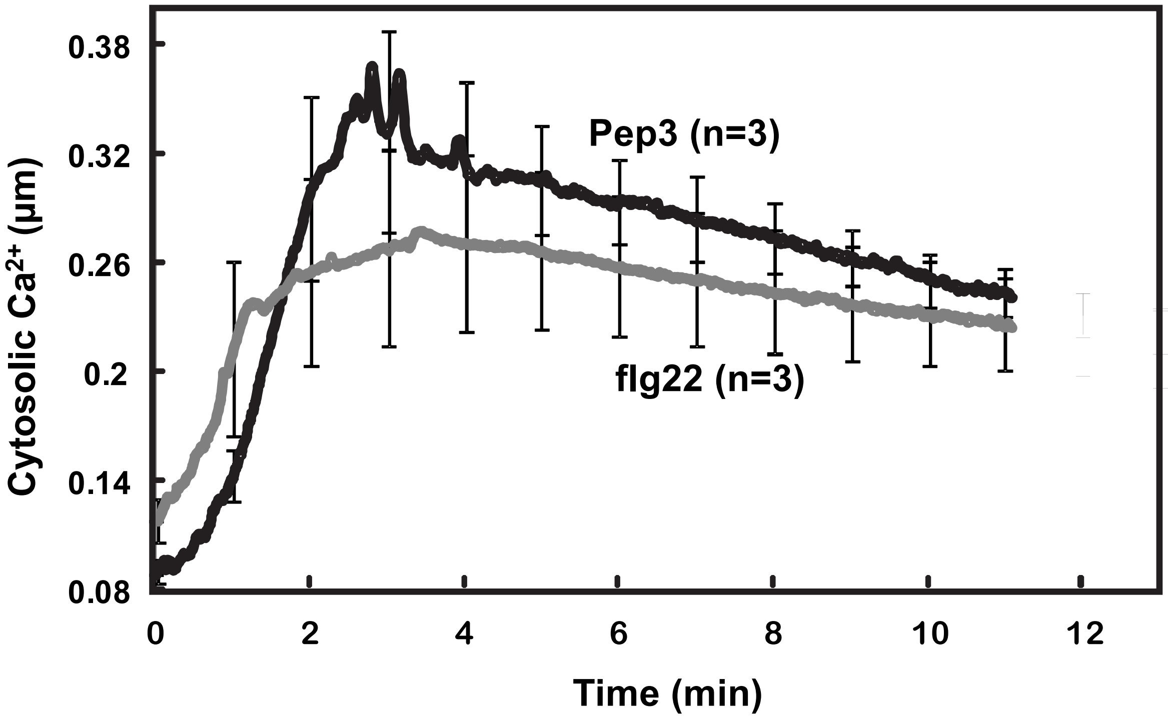

Fig. S1. Flg22- and Pep3-dependent cytosolic Ca^{2+} elevation in wt (Col-aeq) seedlings. Ligand (20 nM flg22 or Pep3) was added at time ‘0’. Results are presented as means (± SE, n=3) calculated at 1 min intervals.

Fig. S2

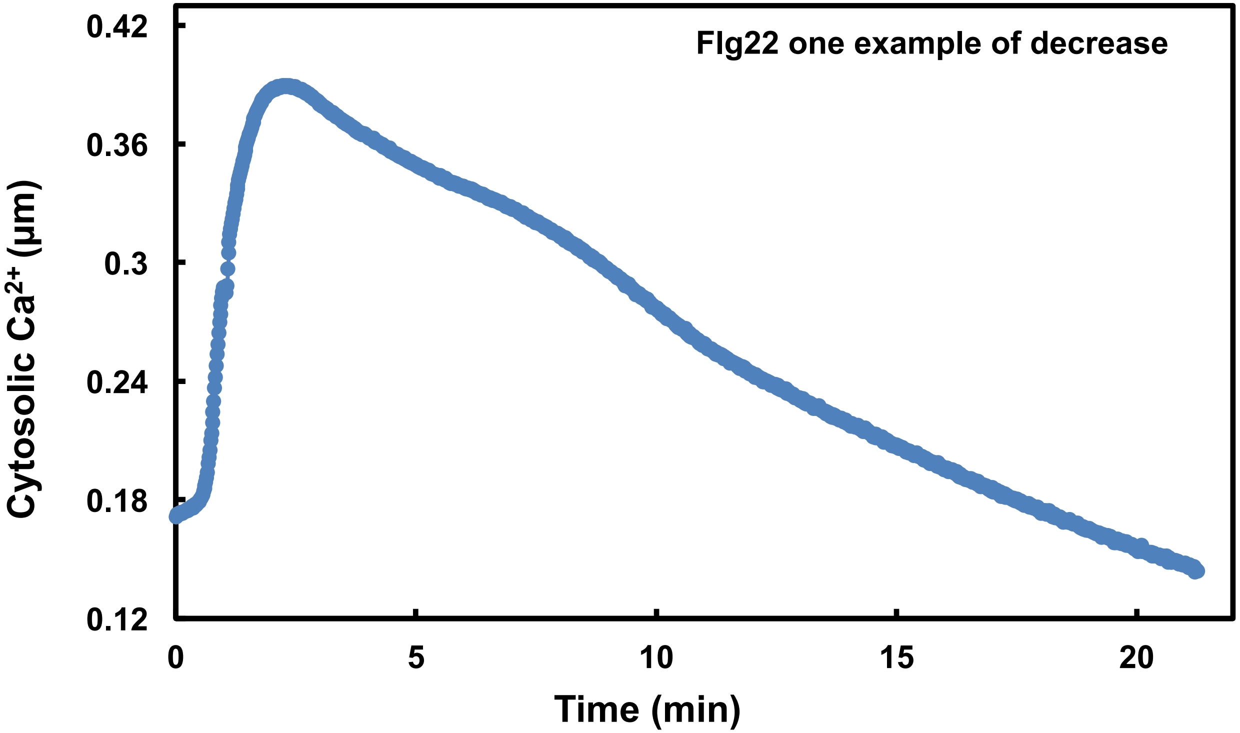

Fig. S2. The flg22-dependent cytosolic Ca^{2+} elevation is transitory. A recording from an individual wt (Col-aeq) seedling exposed to (1 μM) flg22 at time '0' is shown for a 22 min period after ligand addition. This recording is representative of those used for the compiled measurements shown in Fig. 3.
